# Cost-effectiveness analysis of proton beam therapy for treatment decision making in paranasal sinus and nasal cavity cancers in China

**DOI:** 10.1186/s12885-020-07083-x

**Published:** 2020-06-26

**Authors:** Guo Li, Bo Qiu, Yi-Xiang Huang, Jerome Doyen, Pierre-Yves Bondiau, Karen Benezery, Yun-Fei Xia, Chao-Nan Qian

**Affiliations:** 1 Department of Radiation Oncology, Affiliated Cancer Hospital & Institute of Guangzhou Medical University, Guangzhou, Guangdong 510095 P. R. China; 2 State Key Laboratory of Oncology in South China, 651 Dongfeng East Road, Guangzhou, Guangdong 510060 P. R. China; 3 Department of Radiation Oncology, Sun Yat-sen University Cancer Center, Guangzhou, Guangdong 510060 P. R. China; 4 Department of Health Management, Public Health Institute of Sun Yat-sen University, Guangzhou, Guangdong 510000 P. R. China; 5 Department of Radiation Oncology, Antoine Lacassagne Cancer Center, University of Nice-Sophia, 06189 Nice, France; 6 Mediterranean Institute of Proton Therapy, Antoine Lacassagne Cancer Center, University of Nice-Sophia, 06200 Nice, France; 7 Department of Radiation Oncology, Guangzhou Concord Cancer Center, Guangzhou, Guangdong 510045 P. R. China

**Keywords:** Paranasal sinus and nasal cavity cancer, Proton beam therapy, Cost-effectiveness analysis, Intensity-modulated proton radiation therapy, Intensity-modulated photon-radiation therapy, Treatment decision making, Markov model, China

## Abstract

**Background:**

Cost-effectiveness is a pivotal consideration for clinical decision making of high-tech cancer treatment in developing countries. Intensity-modulated proton radiation therapy (IMPT, the advanced form of proton beam therapy) has been found to improve the prognosis of the patients with paranasal sinus and nasal cavity cancers compared with intensity-modulated photon-radiation therapy (IMRT). However, the cost-effectiveness of IMPT has not yet been fully evaluated. This study aimed at evaluating the cost-effectiveness of IMPT versus IMRT for treatment decision making of paranasal sinus and nasal cavity cancers in Chinese settings.

**Methods:**

A 3-state Markov model was designed for cost-effectiveness analysis. A base case evaluation was performed on a patient of 47-year-old (median age of patients with paranasal sinus and nasal cavity cancers in China). Model robustness was examined by probabilistic sensitivity analysis, Markov cohort analysis and Tornado diagram. Cost-effective scenarios of IMPT were further identified by one-way sensitivity analyses and stratified analyses were performed for different age levels. The outcome measure of the model was the incremental cost-effectiveness ratio (ICER). A strategy was defined as cost-effective if the ICER was below the societal willingness-to-pay (WTP) threshold of China (30,828 US dollars ($) / quality-adjusted life year (QALY)).

**Results:**

IMPT was identified as being cost-effective for the base case at the WTP of China, providing an extra 1.65 QALYs at an additional cost of $38,928.7 compared with IMRT, and had an ICER of $23,611.2 / QALY. Of note, cost-effective scenarios of IMPT only existed in the following independent conditions: probability of IMPT eradicating cancer ≥0.867; probability of IMRT eradicating cancer ≤0.764; or cost of IMPT ≤ $52,163.9. Stratified analyses for different age levels demonstrated that IMPT was more cost-effective in younger patients than older patients, and was cost-effective only in patients ≤56-year-old.

**Conclusions:**

Despite initially regarded as bearing high treatment cost, IMPT could still be cost-effective for patients with paranasal sinus and nasal cavity cancers in China. The tumor control superiority of IMPT over IMRT and the patient’s age should be the principal considerations for clinical decision of prescribing this new irradiation technique.

## Background

Paranasal sinus and nasal cavity cancers are rare types of cancer accounting for about 10% of all head and neck cancers [1, 2]. Surgery is only suitable for early-stage diseases (T1-T2N0). For the vast majority of cases, which are already in advanced stage (T2-T4N+) at diagnosis, radiotherapy is the indispensable approach either as post-operative treatment to improve local control or as the radical treatment [3]. Developments of radiation techniques have been a key approach to improve the local control and survival of this disease. With the introduction of conformal targeted radiotherapy and intensity-modulated photon-radiation therapy (IMRT), the 5-year overall survival rate has been improved from 28% in the 1960s to ~ 50% till presently [4].

However, further improvement in the local control of this disease using IMRT seems difficult due to contradiction between its need for high required radical dose to the tumor and the dose limits of the surrounding normal tissues. As a result, the dominance of IMRT is being challenged by a new charged particle radiotherapy mode, termed as proton beam therapy (PBT), which possesses superior dose distribution afforded by protons’ “Bragg peaks” [5, 6]. According to an authoritative systematical review, compared to IMRT, PBT has been found to improve the 5-year overall survival rate of paranasal sinus and nasal cavity cancer from 45.1 to 69.7% [7]. For this reason, paranasal sinus and nasal cavity cancers were included as the “Group 1″ indication of PBT in the “Proton Beam Therapy Model Policy” by the American Society for Radiation Oncology [8].

The advanced form of PBT, intensity-modulated proton radiation therapy (IMPT), has the ability to apply pencil beam scanning technique to scan or “paint” the tumor volume voxel-by-voxel and layer-by-layer, and has undoubtedly become a more advantageous radiotherapy type for paranasal sinus and nasal cavity cancer. However, IMPT is much more expensive than IMRT due to technique complexity of particle therapy. Taking higher capital investment costs and operational costs into account, the cost ratio (IMPT/IMRT) ranges from 3.2 to 4.8 [9]. In china, the incidence of paranasal sinuses and nasal cavity cancer is similar to rest of the world, PBT has been introduced to treat this tumor type for few years. As a developing country with a gross domestic product (GDP) per capita of ~ 10,000 US dollars ($), PBT related costs are not yet covered by Chinese public medical insurance due to limited medical resources. Hence, cost-effectiveness analysis (CEA) is urgently-needed in clinical decision making for the appropriate radiotherapy mode [10].

The Markov model is a stochastic model that can track the natural process of chronic disease (i.e. tumor) by multiple cycles of operation and has become an ideal method to assess the cost-effectiveness of cancer treatment in a long-term period [11]. In this study, we designed a 3-state Markov model to evaluate the cost-effectiveness of PBT with Chinese settings and to facilitate the decision making for paranasal sinus and nasal cavity cancer treatment.

## Methods

### Model design

The TreeAge Pro 2018 software (TreeAge Software, Williamstown, MA) was used for building and analyzing the Markov model. A decision tree combined with two 3-state Markov models were designed to evaluate the cost-effectiveness of IMPT in comparison to that of IMRT for paranasal sinus and nasal cavity cancer. We used a base case of 47-year-old (median age of this cancer type in China) to represent the average Chinese paranasal sinus and nasal cavity cancer patient [12]. The 2 compared treatment strategies were similar except for the radiation technique (IMPT vs. IMRT), which included a radical radiotherapy and 3 cycles of concurrent chemotherapy. Other clinical outcomes, including irradiation-induced acute and late toxicities, were assumed to be identical between the two strategies. The model input information for the base case was presented in Table 1.

**Table 1:** Model information of the base case used and probabilistic sensitivity analysis of the parameters

Parameters	Input Information	90% CI in PSA^**a**^	Distribution^**b**^	Source
**Base case**				
Age (year-old)^c^	47			
Tumor stage	T3N1M0, Stage III			
**Evaluated radiotherapy modes**	IMPT vs. IMRT			
**Cost ($)**				
IMPT	50,000	37,258.3–62,775.4	Normal	SPHIC
IMRT	12,000	10,723.2 - 13,287.5	Normal	SYSUCC
Concurrent chemotherapy	5000	3710.4 - 6284.7	Normal	SYSUCC
Follow-up / year	1000	872.1–1127.8	Normal	SYSUCC
Palliative therapy / year	5000	3724.0 - 6269.1	Normal	SYSUCC
**Utilities (QALYs)**				
No cancer	0.94	0.822–1	Beta	Ward MC et al. [13]
Alive with cancer	0.47	0.343–0.598	Beta	Noel CW et al. [14]
Death	0			
**Transition probabilities**				
IMPT eradicating cancer	0.9	0.833–0.957	Beta	Samir H Patel et al. [7]
IMRT eradicating cancer	0.73	0.665–0.792	Beta	Dulguerov P et al. [4], Samir H Patel et al. [7]
“no cancer” - “alive with cancer”	0.1(2nd-3rd year); 0.05(4th–5th year); 0.01(6th -10th year); 0(6th -10th year)			Dulguerov P et al. [4], Samir H Patel et al. [7]
“alive with cancer” - “death”	0.3	0.237–0.365	Beta	Dulguerov P et al. [4], Samir H Patel et al. [7]
**Risk of non-cancer death**	2016 Life Tables			Social Security Administration [15]
**Model set-up**				
Cycle length	1-year			
No. of cycles^d^	30			
Discount rate / year	3%			

*CI* confidence interval, *PSA* probabilistic sensitivity analysis, *IMPT* intensity modulated proton radiation therapy, *IMRT* intensity modulated photon-radiation therapy, *$* US dollars*, QALY* quality adjusted life year, *SPHIC* Shanghai proton and heavy Ion Center, *SYSUCC* Sun Yat-sen university cancer center

^a^Probabilistic sensitivity analysis was applied to determine 90% CI for model parameters by running over 50,000 iteration trials

^b^The utilities and probabilities were tested with beta distributions and the costs were tested with uniform distributions

^c^Median age of paranasal sinus and nasal cavity cancer in China [12]

^d^Markov models were to be cycled 30 times to evaluate the treatment outcomes over a 30-year time period for the base case until 77-year-old (Chinese life expectancy)

### States and transition probabilities

**Fig. 1:**
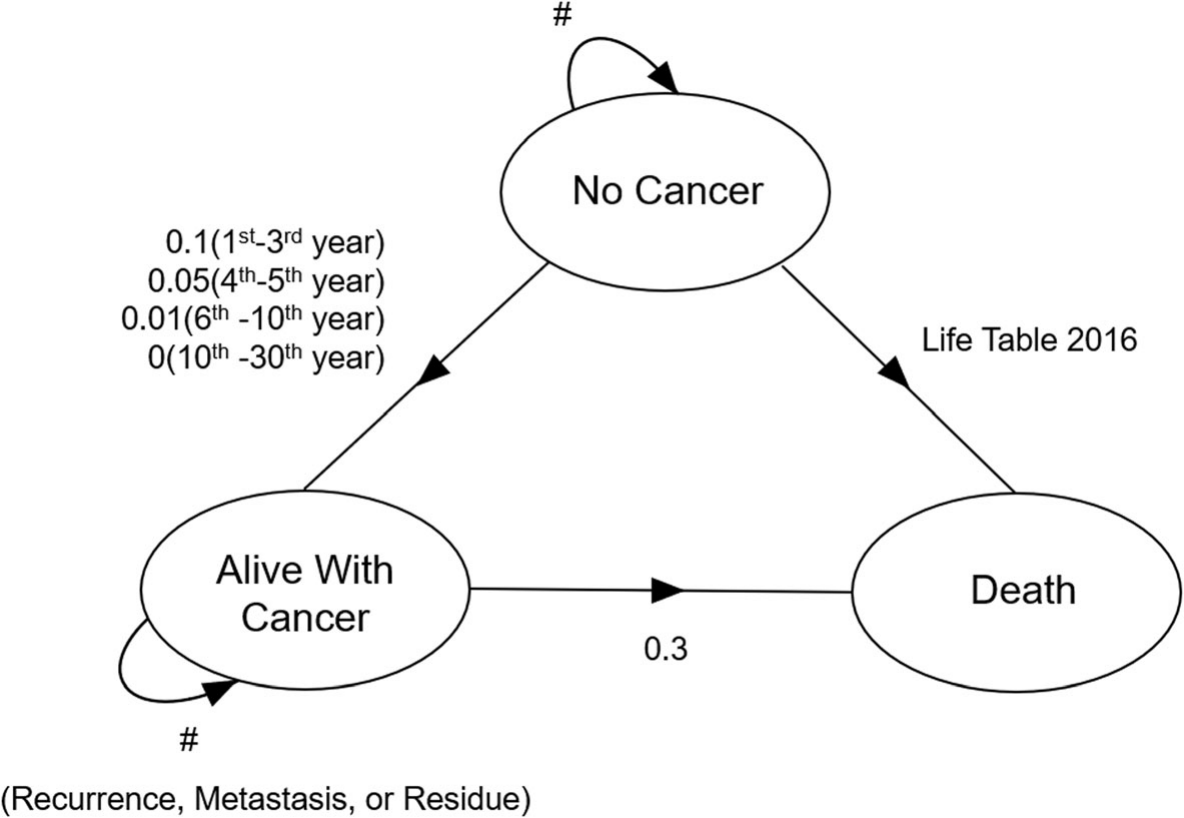
Diagram of transition states in Markov model. In the designed Markov model, we used 3 transition states of “no cancer”, “alive with cancer” and “death” to simulate the disease process of the patient after radiotherapy. 1-year cycle length was used, and the Markov model will be cycled until the patient’s 77-year-old (Chinese life expectancy). For each cycle, if the patient was in the state of “no cancer”, s/he might stay in the state of “no cancer”, develop into the state of “alive with cancer” or develop into the state of “death”; If the patient was in the state of “alive with cancer”, s/he might stay in the state of “alive with cancer” or develop into the state of “death”; If the patient was in the absorbing state “death”, the loop operation would be terminated

States and transition probabilities of the Markov model were illustrated in Fig. 1. Three states including “no cancer”, “alive with cancer” and “death” were used to simulate the disease process of this patient after radiotherapy. We assumed that IMPT and IMRT had a respective probability of 0.9 and 0.73 for eradicating the tumor [4]. If the cancer was totally eradicated after radiotherapy (defined as “complete response” by magnetic resonance imaging of the head and neck 3 month after radiotherapy), the initial state would be “no cancer”; and if not, the initial state would be “alive with cancer” (recurrence, metastasis or residue). The transition probability from “alive with cancer” to cancer-related “death” was 0.3 per year, the probability from “no cancer” to “alive with cancer” was assumed as 0.1 for the 1st to 3rd year after radiotherapy, 0.05 for the 4th and 5th years, 0.01 for the 6th to 10th year, and 0 for the following 20 years [7]. The risk of natural non-cancer caused death was calculated based on the 2016 Life Tables of United States [15]. Costs and quality-adjusted life-years (QALYs) were discounted at an annual rate of 3% [16]. A 1-year cycle length was used and the Markov models were to be cycled 30 times to evaluate the treatment outcomes over a 30-year time period for the base case until the estimated 2020 Chinese life expectancy (77-year-old) [17].

### Cost and utilities

The costs of treatment were calculated based on casual clinical prescriptions to reflect similar costs as that of daily practice in a Chinese hospital. All costs were adjusted to $, using a Sino-US exchange rate of $1 = 6.93 RMB (January 23, 2020). The cost of IMPT and IMRT were thereby estimated as being $50,000 and $12,000 respectively for simulating a 32 fractions to a total dose of 70 Gy. The cost of concurrent chemotherapy was assumed as $5000 (simulating 3 cycles of 80–100 mg/m2 cisplatin bolus injection delivered on day-1, − 21 and − 42 of the radiotherapy). The follow-up cost per year was assumed as $1000 (including hematologic and biochemistry profiles, nasopharyngeal fiberoptic endoscope examination, magnetic resonance imaging of head and neck, chest radiography and abdominal ultrasonography); the cost of palliative therapy per year was assumed as $5000 (simulating 8 cycles of oral palliative chemotherapy with 5-fluorouracil). For patients in the “no cancer” state, the incremental cost per year was only the follow-up cost. For patients in the “alive with cancer” state, the incremental cost per year included follow-up cost and the cost of palliative chemotherapy.

The utilities were adjusted to QALYs using health state utility values (HSUV) derived from previously published studies for head and neck cancer. HSUV can be interpreted as the strength of preference for a given health state on a cardinal scale anchored at 0 (‘death’) and 1 (‘full health’). In this model, initial HSUV after radiotherapy in the first year was assumed as 0.47, the same as the HSUV of “alive with cancer” for a progressive disease with the disutility caused by anticancer treatment [13]. HSUV of “no cancer” was assumed as 0.94 for the situation of no cancer and no treatment after radiotherapy [14, 18].

### Sensitivity analysis and Monte Carlo simulation

Probabilistic sensitivity analysis was applied to illustrate the robustness of the model in light of a joint uncertainty for model parameters by running over 50,000 iteration trials. Tornado diagram was used to evaluate the influence of the variation of each parameter to the incremental cost-effectiveness ratio (ICER). One-way sensitivity analyses were conducted to identify thresholds for the expected values at which IMPT can be cost-effective. Monte Carlo simulation (50,000 trials) was applied to show trials distribution of the two strategies to determine which would be the recommended strategy.

### Outcomes measurement

Overall survival was defined as time interval between the end of the radiotherapy and death from any cause. Disease-free survival was defined as the time interval between the end of the radiotherapy and first cancer progression or death from any cause. The outcome measure of the model was the ICER which represented the ratio of the difference in costs to the difference in effectiveness (incremental cost / incremental effectiveness) between IMPT and IMRT. A strategy is deemed cost-effective by comparing the ICER with an established societal willingness to pay (WTP). According to the World Health Organization guidelines, a strategy is defined as cost-effective if the ICER is below three times the GDP per capita, so $30,828 / QALY (3 times Chinese GDP per capita in 2019) was applied as the WTP threshold of China in this study [19, 20].

## Results

### Model robustness verification

Probabilities results of Markov cohort analyses for the two strategies were shown in Additional file 1: Figure S1. The 2-, 5- and 10-year overall survival rates were 83.5, 51.9 and 43.9% for the IMRT strategy; and 91.6, 72.4 and 53.5% for the IMPT strategy. The 2-, 5- and 10-year disease-free survival rates were 84.0, 63.1 and 46.1% for the IMRT strategy; and 92.2, 73.8 and 56.2% for the IMPT strategy. Overall survival data of IMPT strategy and IMRT strategy were similar with the previous outcomes (detailed in Additional file 2: Figure S2).

**Fig. 2:**
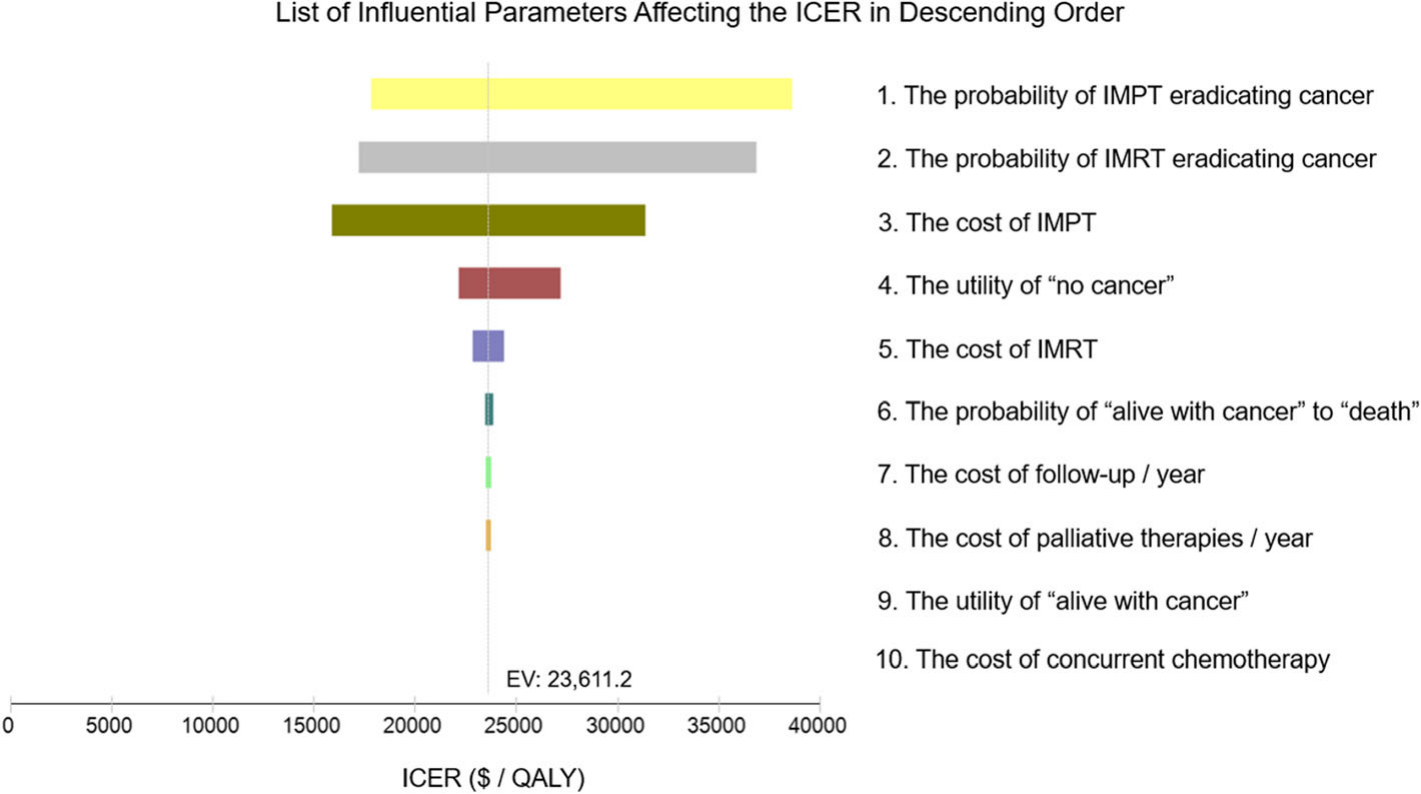
Tornado diagram analysis of influential parameters affecting the incremental cost-effectiveness ratio. The Tornado diagram (one-way sensitivity analysis) demonstrated the range of incremental cost-effectiveness ratio (ICER) when varying each parameter individually. Influential parameters were listed in descending order according to their abilities of affecting the ICER over the variation of their 90% confidence interval. This demonstrated that the model was just sensitive to the probability of IMPT eradicating cancer, the probability of IMRT eradicating cancer and the cost of IMPT, which did correspond to our model design. IMPT, intensity modulated proton radiation therapy; IMRT, intensity modulated photon-radiation therapy; EV, expected value; QALY, quality adjusted life year; $, US dollars

Probabilistic sensitivity analysis was performed to evaluate the uncertainty of each parameter simultaneously with 50,000 iterations. The 90% confidence interval and distributions of the parameters were listed in Table 1. The Tornado diagram identified that the top 3 parameters influencing the ICER of the base case were the probability of IMPT eradicating cancer, the probability of IMRT eradicating cancer and the cost of IMPT. The other parameters had only a minor impact on the ICER (Fig. 2).

### Cost-effectiveness of the base case

Overall, the QALYs in the IMPT strategy were higher than those in the IMRT strategy (10.18 QALYs vs. 8.53 QALYs). By model calculation, IMPT strategy provided an additional 1.65 QALYs at an additional cost of $38,928.7, and ICER of IMPT compared with IMRT was $23,611.2 / QALY, below the WTP threshold of China ($30,828 / QALY). Hence, IMPT was cost-effective for the base case representing the average Chinese paranasal sinus and nasal cavity cancer patient. Monte Carlo simulations showed that IMPT was the recommended strategy in 13.5% of trials at the WTP of China. Additional file 3: Figure S3 showed the cost-effectiveness scatterplot comparing IMPT to IMRT.

### Cost-effective scenarios with the base case setting

For the base case, the threshold values of the top 3 parameters influencing ICER at which IMPT could be considered as cost-effective were respectively identified by one-way sensitivity analyses with 3 different WTP thresholds ($30,828 / QALY, $50,000 / QALY and $100,000 / QALY) (Table 2). The cost-effective scenarios of IMPT only existed in the following independent conditions: the probability of IMPT eradicating cancer was ≥0.859 at the WTP of China (≥ 0.809 at a WTP of $50,000 / QALY, ≥ 0.769 at a WTP of $100,000 / QALY); the probability of IMRT eradicating cancer ≤0.771 at the WTP of China (≤ 0.821 at a WTP of $50,000 / QALY, ≤ 0.861 at a WTP of $100,000 / QALY); or the cost of IMPT ≤ $61,899 at the WTP of China (≤ $93,508.3 at a WTP of $50,000 / QALY, ≤ $175,945.3 at a WTP of $100,000 / QALY).

**Table 2:** One-way sensitivity analysis of influential parameters affecting the incremental cost-effectiveness ratio of the base case

Parameters^**a**^	Base Value	Expected Value	With 3 Different WTP Thresholds		
			$30,828 / QALY^**b**^	$50,000 / QALY	$100,000 / QALY
The probability of IMPT eradicating cancer	0.9	Minimum	0. 859	0.809	0.769
The probability of IMRT eradicating cancer	0.73	Maximum	0.771	0.821	0.861
The cost of IMPT ($)	50,000	Maximum	61,899	93,508.3	175,945.3

*IMPT* intensity modulated proton radiation therapy, *IMRT* intensity modulated photon-radiation therapy, *WTP* willingness to pay, *$* US dollars, *QALY* quality-adjusted life-year

^**a**^The top 3 parameters influencing the incremental cost-effectiveness ratio identified by Tornado diagram

^**b**^The WTP threshold of China

### Cost-effectiveness and patient age

**Fig. 3:**
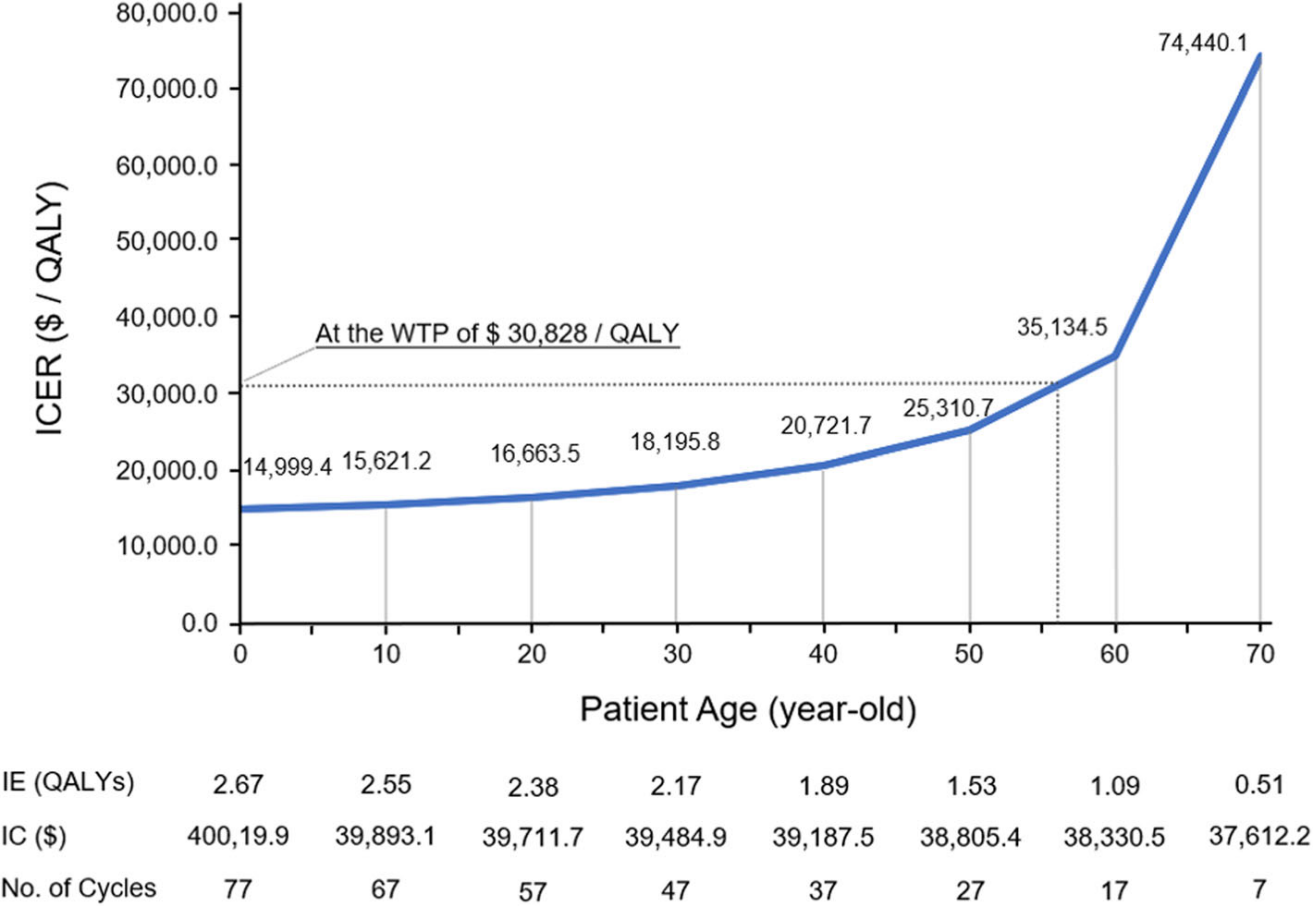
Cost-effectiveness of IMPT in patients of different age levels. Incremental cost (IE), incremental effectiveness (IC) and incremental cost-effectiveness ratio (ICER) were evaluated by stratified analyses for patients of 0,10, 20, 30, 40, 50, 60, 70-year-old levels to the endpoint of the Chinese life expectancy (77-year-old). IMPT, intensity modulated proton radiation therapy; IMRT, intensity modulated photon-radiation therapy; QALY, quality adjusted life year; WTP: willingness to pay; $, US dollars

Stratified analyses evaluated the cost-effectiveness of IMPT in patients of different age levels, as shown in Fig. 3**.** The ICERs were $14,999.4/QALY, $15,621.2/QALY, $16,663.5/QALY, $18,195.8/QALY, $20,721.7/QALY, $25,310.7/QALY, $35,134.5/QALY, $74,440.1/QALY for 0,10, 20, 30, 40, 50, 60, 70-year-old levels, respectively. One-way sensitivity analyses identified that IMPT was cost-effective in patients ≤56-year-old at the WTP of China, in patients ≤66-year-old at a WTP of $50,000 / QALY, and in patients ≤71-year-old at a WTP of $100,000 / QALY.

## Discussion

According to current data from the Particle Therapy Co-operative Group, only 1 proton center is in operation for the 1.4 billion Chinese population in China, and at least 10 new proton centers will start to treat patients in next 3 years due to huge demands [21]. Considering high treatment cost as a limiting factor hindering the use of PBT, cost-effectiveness evaluation is of urgent need for the treatment decision making when PBT become more available in the near future. To our knowledge, this is the first study on CEA modeling of PBT which was designed specifically for China.

Due to deficiency of valid data and lacking of a uniform model pattern, effective and reliable CEA studies of PBT are rare worldwide [22]. Some classical studies such as a 3-state Markov model designed for breast cancer, which focused on the advantages of IMPT in reducing the incidence of irradiation-induced coronary heart disease, concluded that IMPT was cost-effective for patients with 1 cardiac risk factors when photon are unable to achieve mean dose of heart < 5 Gy [23]. Another 6-state Markov model designed for Stage IVa oropharynx cancer, which was based on a hypothesis that IMPT could make a 25% reduction of xerostomia, dysgeusia and the need for gastrostomy tube, concluded that IMPT was cost-effective only in younger patients who could benefit from profound reductions of long-term morbidity [24]. These two Markov models focused on the late toxicities’ reduction of IMPT and could hardly be popularized to the tumor types for which IMPT could obviously improve tumor control compared with IMRT. Due to the anatomical location and a relatively low radiosensitivity of paranasal sinus and nasal cavity cancer, the advantage of IMPT compared with IMRT mainly lies in the improvement of tumor control, and no significant reduction of acute and late toxicities was found in report of Samir H Patel et al. [7]. Therefore, toxicities were assumed to be identical between the two strategies in our model building. This 3-state Markov model could be easily applied to the tumor types with similar behavior.

For validating model robustness, probabilistic sensitivity analyses were used to evaluate the uncertainties of parameters by their value variations. The Tornado diagram showed that only the probability of IMPT eradicating cancer, the probability of IMRT eradicating cancer and the cost of IMPT had significant impact on ICER. These results did correspond to our model design in which we focused on the advantage of IMPT in improving tumor control compared with IMRT so that the difference in probabilities for eradicating tumor between the two strategies would be the source of the difference in effectiveness and cost. Furthermore, the 2-, 5- and 10-year overall survival rates of the model were calculated and found to be within the ranges of previously published survival data of paranasal sinus and nasal cavity cancer, which demonstrated that our proposed model did abide with the natural process of this disease.

The specific scenarios that may benefit from IMPT were further analyzed, and results of CEA further supported that the application of IMPT for this type of tumor was reasonable from the perspective of the daily Chinese clinical practice. For the base case, IMPT should at least achieve a 0.859 probability of eradicating cancer. This probability is readily achieved as the actual 5-year locoregional control rate of paranasal sinus and nasal cavity cancer treated by PBT is 89.5% [7]. On the other hand, IMRT could hardly obtain the expected eradication probability of 0.771 in general. Hence, the specific scenarios in favor of IMPT in clinical practice would be when IMRT is considered as inability to well eradicate the tumor. The threshold of maximum cost of IMPT ($52,163.9) demonstrated the current price ($50,000) as reasonable considering the economic situation of China. Stratified analyses of different age levels showed that IMPT was considered as cost-effective only in patients ≤56-years old using the current WTP threshold of China. And this age threshold would increase with the growth of WTP threshold (the growth of GDP per capita) and the cost reduction of IMPT. With an assumption of a 20% cost reduction (with a cost of IMPT $40,000), IMPT could be cost-effective in patients ≤63-year-old at the WTP of China, in patients ≤69-year-old at a WTP of $50,000 / QALY, and in patients ≤72-year-old at a WTP of $100,000 / QALY. Therefore, we presume that IMPT would be more recommended in the future.

There are three main limitations to this model. First, the HSUVs applied in our model were derived from previously published studies for head and neck cancer because of the lack of specific HSUVs for paranasal sinus and nasal cavity cancer. As previously reported, the overall mean HSUV of head and neck cancer survivors after radiotherapy was consistently considered as 0.7 [25, 26]. In this study, the survivors after radiotherapy had two different states, namely as “alive with cancer” and “no cancer”. Therefore, we chose 0.47 as HSUV of “alive with cancer” for describing the utility of salvage treatment relative to chemotherapy, as reported by Ward MC et al. [13]; and HSUV of “no cancer” was assumed as 0.94 for describing the situation of no cancer and no need of treatment, as reported by Noel CW et al. [14]. Second, our CEA results indicated that the benefits obtained from IMPT (the improvement of tumor control in comparison with IMRT) was a principal consideration for the clinical decision of prescribing IMPT, but the current analyses with base case setting had not involved evaluating such benefits of an individual patient. So, we plan to use model-based approaches (such as tumor control probability model) to estimate personal benefits from IMPT for supporting individualized decision making in the future [27]. Third, due to the current deficiency of valid data about adverse events between IMPT and IMRT for paranasal sinus and nasal cavity cancers, such as acute/late toxicities and hospital admission rates during treatment, the effectiveness gain of IMPT in reducing these adverse events were not taken into the effectiveness calculation. We assumed that this limitation might be the cause of the negative results in trials of Monte Carlo simulations (only 13.5% of trials favored IMPT to IMRT), which were more susceptible to long-term differences [28]. With more data from the underway clinical trials evaluating toxicities and other clinical outcomes (such as NCT00797498 (photon/proton radiation therapy for cancers of the nasal cavity and/or paranasal sinuses)), the advantages of IMPT for this tumor type would be further evaluated, and IMPT might be more recommended with respect to patient’s life quality after radiotherapy.

## Conclusions

Cost-effectiveness is a pivotal consideration for decision making of PBT in developing countries. To the best of our knowledge, this is the first CEA study of PBT designed for the specific situation of China. The 3-state Markov model designed for paranasal sinus and nasal cavity cancer was found to be practical, reliable and representative and could be easily applied to CEA studies of other similar tumor types. Our CEA results further evinced that IMPT was cost-effective for paranasal sinus and nasal cavity cancer treatment in the Chinese settings. The probability of IMPT eradicating cancer, the probability of IMRT eradicating cancer, the cost of IMPT and the patient’s age were found to be decisive components influencing ICER. Although our findings point that IMPT was cost-effective for paranasal sinus and nasal cavity cancer in patients ≤56-year-old, as the growth of WTP threshold and the cost reduction of IMPT, IMPT is expected to become more recommended in the future with respect to cost-effectiveness.

## Supplementary information

**Additional file 1: Figure S1.** Markov probabilities analyses for the base case. **a.** Markov probabilities analyses in IMPT strategy. **b.** Markov probabilities analyses in IMRT strategy. Markov cohort analyses were applied to calculate the state probabilities for the base case (47-year-old) in both intensity modulated proton radiation therapy (IMPT) strategy and intensity modulated photon-radiation therapy (IMRT) strategy.

**Additional file 2: Figure S2.** Overall survival data of IMPT strategy and IMRT strategy in comparison with the previous outcomes. The 5-year overall survival rates of intensity modulated proton radiation therapy (IMPT) and intensity modulated photon-radiation therapy (IMRT) strategy were within the ranges of 38–60% and 52–85% as described in a previous report of Samir H Patel et al. [7]; The 2-, 5- and 10-year overall survival rates of IMRT strategy were in comparison with the previous outcomes of 75, 60 and 47% reported by Dulguerov P et al. [4].

**Additional file 3: Figure S3.** Incremental cost-effectiveness scatter plot and strategy selection chart in trials of Monte Carlo simulations. a. Trials distribution in incremental cost-effectiveness scatter plot. b. Strategy selection chart. Monte Carlo simulation (with 50,000 trials) was performed for the base case of 47-year-old at the WTP of $30,828 / QALY. **a,** each point represented 1 of those simulations and was charted at the simulation’s resultant incremental cost versus incremental effectiveness of IMPT compared with IMRT. **b,** strategy selection from the perspective of net benefit demonstrated that only 13.5% of trials favored IMPT to IMRT. $, US dollars; IMPT, intensity modulated proton radiation therapy; IMRT, intensity modulated photon-radiation therapy; QALY, quality adjusted life year; WTP: willingness to pay.

## Data Availability

The datasets used and analyzed during the current study are available from the corresponding author on reasonable request.

## Abbreviations

IMPT: Intensity-modulated proton radiation therapy
IMRT: Intensity-modulated photon-radiation therapy
PSA: Probabilistic sensitivity analysis
ICER: Incremental cost-effectiveness ratio
WTP: Willingness-to-pay
GDP: Gross domestic product
$: US dollars
QALY: Quality-adjusted life year
PBT: Proton beam therapy
CEA: Cost-effectiveness analysis
SPHIC: Shanghai proton and heavy Ion Center
SYSUCC: Sun Yat-sen University Cancer Center
HSUV: Health state utility values
IC: Incremental cost
IE: Incremental effectiveness
