# Supplementary figures and images for: Cost-effectiveness analysis of proton beam therapy for treatment decision making in paranasal sinus and nasal cavity cancers in China
Source: BMC Cancer. 2020 Jun 26;20:599. doi: 10.1186/s12885-020-07083-x (PMC7320568; doi:10.1186/s12885-020-07083-x)

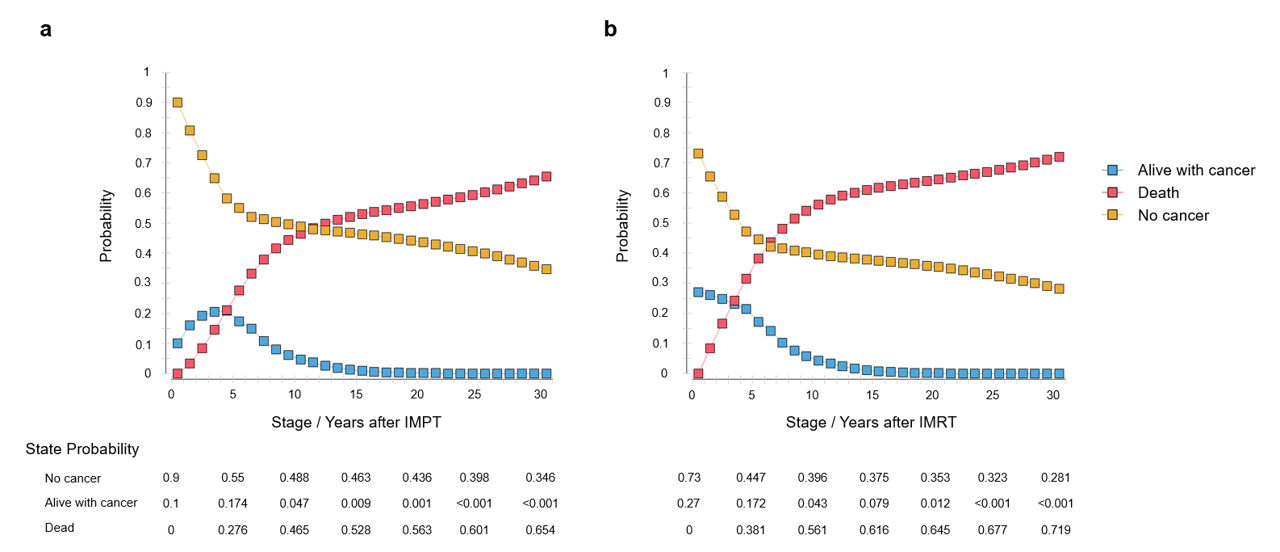

Supplement: Supplementary file 1 — Additional file 1: Figure S1. Markov probabilities analyses for the base case. a. Markov probabilities analyses in IMPT strategy. b. Markov probabilities analyses in IMRT strategy. Markov cohort analyses were applied to calculate the state probabilities for the base case (47-year-old) in both intensity modulated proton radiation therapy (IMPT) strategy and intensity modulated photon-radiation therapy (IMRT) strategy. [file 12885_2020_7083_MOESM1_ESM.tif]

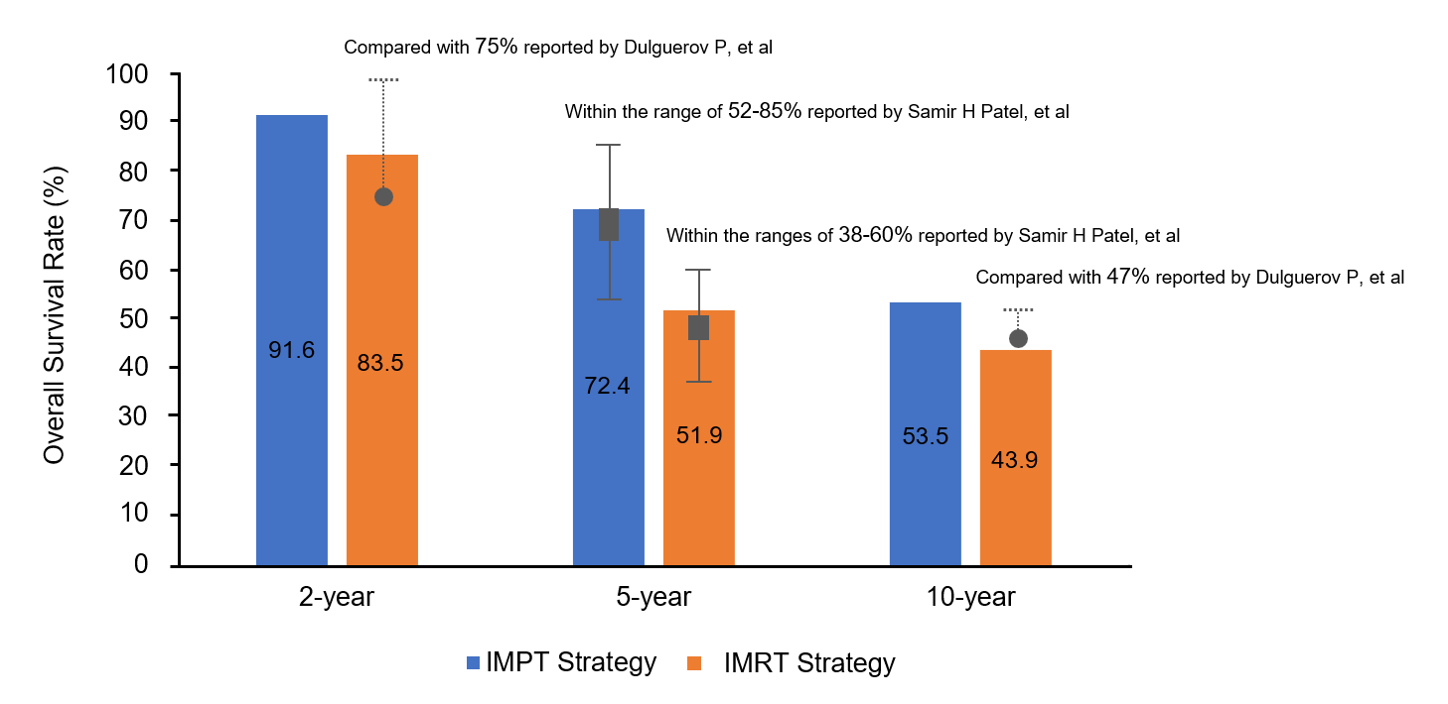

Supplement: Supplementary file 2 — Additional file 2: Figure S2. Overall survival data of IMPT strategy and IMRT strategy in comparison with the previous outcomes. The 5-year overall survival rates of intensity modulated proton radiation therapy (IMPT) and intensity modulated photon-radiation therapy (IMRT) strategy were within the ranges of 38–60% and 52–85% as described in a previous report of Samir H Patel et al. [7]; The 2-, 5- and 10-year overall survival rates of IMRT strategy were in comparison with the previous outcomes of 75, 60 and 47% reported by Dulguerov P et al. [4]. [file 12885_2020_7083_MOESM2_ESM.tif]

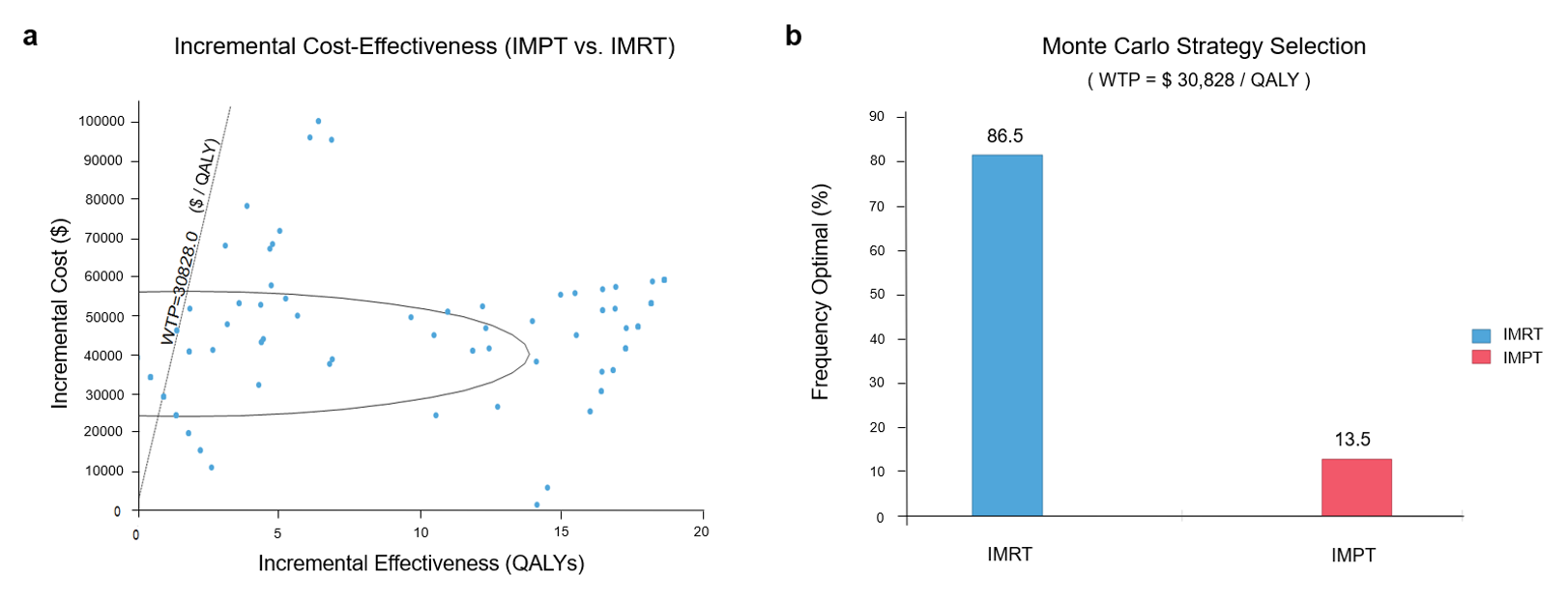

Supplement: Supplementary file 3 — Additional file 3: Figure S3. Incremental cost-effectiveness scatter plot and strategy selection chart in trials of Monte Carlo simulations. a. Trials distribution in incremental cost-effectiveness scatter plot. b. Strategy selection chart. Monte Carlo simulation (with 50,000 trials) was performed for the base case of 47-year-old at the WTP of $30,828 / QALY. a, each point represented 1 of those simulations and was charted at the simulation’s resultant incremental cost versus incremental effectiveness of IMPT compared with IMRT. b, strategy selection from the perspective of net benefit demonstrated that only 13.5% of trials favored IMPT to IMRT. $, US dollars; IMPT, intensity modulated proton radiation therapy; IMRT, intensity modulated photon-radiation therapy; QALY, quality adjusted life year; WTP: willingness to pay. [file 12885_2020_7083_MOESM3_ESM.tif]
